# Microcirculatory changes during open label magnesium sulphate infusion in patients with severe sepsis and septic shock

**DOI:** 10.1186/1471-2253-11-12

**Published:** 2011-06-14

**Authors:** Andrius Pranskunas, Namkje AR Vellinga, Vidas Pilvinis, Matty Koopmans, E Christiaan Boerma

**Affiliations:** 1 Department of Intensive Care Medicine, Hospital of Lithuanian University of Health Sciences, A.Mickeviciaus g.9 Kaunas, LT 44307, Lithuania; 2 Department of Intensive Care Medicine, Medical Center Leeuwarden, Henri Dunantweg 2, Leeuwarden, 8901 BR, the Netherlands; 3 Department of Translational Physiology, Academic Medical Center, Amsterdam, the Netherlands

## Abstract

**Background:**

Microcirculatory alterations play a pivotal role in sepsis and persist despite correction of systemic hemodynamic parameters. Therefore it seems tempting to test specific pro-microcirculatory strategies, including vasodilators, to attenuate impaired organ perfusion. As opposed to nitric oxide donors, magnesium has both endothelium-dependent and non-endothelium-dependent vasodilatory pathways.

**Methods:**

In a single-center open label study we evaluated the effects of magnesium sulphate (MgS) infusion on the sublingual microcirculation perfusion in fluid resuscitated patients with severe sepsis and septic shock within the first 48 hours after ICU admission. Directly prior to and after 1 hour of magnesium sulphate (MgS) infusion (2 gram) systemic hemodynamic variables, sublingual SDF images and standard laboratory tests, were obtained.

**Results:**

Fourteen patients (12 septic shock, 2 severe sepsis) with a median APACHE II score of 20 were enrolled. No significant difference of the systemic hemodynamic variables was found between baseline and after MgS infusion. We did not observe any significant difference pre and post MgS infusion in the primary endpoint microvascular flow index (MFI) of small vessels: 2.25(1.98-2.69) vs. 2.33(1.96-2.62), p = 0.65. Other variables of microcirculatory perfusion were also unaltered. In the overall unchanged microvascular perfusion there was a non-significant trend to an inverse linear relationship between the changes of MFI and its baseline value (y = -0.7260 × + 1.629, r^2 ^= 0.270, p = 0.057). The correlation between baseline Mg concentrations and the change in MFI pre- and post MgS infusion was non-significant (r_s _= -0.165, p = 0.67).

**Conclusions:**

In the setting of severe sepsis and septic shock sublingual microcirculatory alterations were observed despite fulfillment of sepsis resuscitation guidelines. After infusion of a limited and fixed dose of MgS, microcirculatory perfusion did not improve over time.

**Trial registration:**

ClinicalTrials.gov NTC01332734.

## Background

Microcirculatory dysfunction in sepsis is crucial in the pathogenesis of organ dysfunction and consists of a cascade of mechanisms, which involves many cells, such as endothelial cells, smooth muscle cells, red blood cells and leukocytes [1]. New microcirculatory imaging techniques, such as orthogonal polarization spectral (OPS) imaging [2] and its technical successor sidestream dark field (SDF) imaging [3], have allowed direct observation of the microcirculation at the bedside. De Backer et al. [4] were the first to report that the observed microcirculatory alterations in sepsis were associated with morbidity and mortality, irrespective of correction of global hemodynamic variables [5].

Therefore, other strategies that aim to attenuate microcirculatory dysfunction directly at the level of the microcirculation, have been tested. According to Poiseuille's Law, blood flow through a vessel is directly proportional to the driving pressure along the vessel and its radius to the fourth power, and inversely proportional to the length of the vessel and the dynamic blood viscosity. Theoretically, vasodilators should be able to increase blood flow via a change in vessel diameter at the entrance of the microcirculation and recruit microcirculatory perfusion in volume resuscitated patients [6]. Stimulation of endothelium-dependent vasodilation by topical application of acetylcholine was effective in the recruitment of shut-down capillaries in septic patients, thus challenging the concept of vasoplegia [7]. It suggested that endothelial vasodilatory response is intact in sepsis and microvascular thrombosis is not the predominant factor in the observed decrease in microcirculatory blood flow. Thus, an ideal agent to recruit the microcirculation in sepsis should have endothelium modulator and vasodilator characteristics, such as a nitric oxide (NO) donor [8,9]. However, a recent randomized clinical trial could not confirm the previous observation, that nitroglycerin improved microvascular perfusion in an open label setting [10].

Studies with magnesium (Mg) and magnesium sulphate (MgS) have shown a peripheral (predominantly arteriolar) vasodilator effect with preserved cardiac function, within a large safety margin [11-13], not only indirectly by an endothelium-dependent release of NO [14,15] but also directly via its ability to induce endothelium-independent vasodilation by a direct action on vascular smooth muscle as a calcium competitor. In addition to vasodilatory effects, infusion of MgS is also associated with other potential pro-microcirculatory effects, such as an increase in red blood cell deformability, reduction of platelet aggregation, anti-inflammatory effects and maintenance of endothelial integrity [16-19].

However, up to date the effect of MgS infusion on microvascular perfusion in septic patients, is unknown. Our study aims to test the hypothesis that MgS infusion may improve sublingual microcirculatory perfusion in patients with severe sepsis and septic shock.

## Methods

### Setting

The study was performed during an 8-months period in 2010 in a closed-format 15-bed mixed ICU in a university hospital. It was designed as a prospective, single-centre, open-label trial. Patients were included with less than 48 hrs duration of severe sepsis or septic shock as defined by the International Sepsis Definition Conference [20]. Exclusion criteria were pregnancy, oral bleeding, age < 18 years, liver cirrhosis, acute arrhythmias, advanced malignancy or a mean arterial pressure (MAP) < 65 mmHg refractory to vasopressors. The study was carried out in compliance with the Helsinki Declaration and approved by the local ethics committee (Kauno Regioninis Biomedicininiu Tyrimu Etikos Komitetas, 201003.03 nr BE-2-6) and informed consent was obtained from each patient, according to applicable laws.

### Protocol and data collection

All patients were equipped with an arterial line and central venous catheter or a pulmonary artery catheter (TD-I, B.Braun Medical, Bethlehem, USA). Prior to the start of MgS infusion, all patients followed a strict protocol to optimize systemic hemodynamic parameters in accordance with international guidelines for management of sepsis [21]. Patients were resuscitated such that mean MAP was ≥ 65 mm Hg, central venous pressure no less than 8 mm Hg and mixed venous saturation (SvO_2_) ≥ 65% or central venous saturation (S(c)vO_2_) ≥ 70%. Red blood cell transfusion trigger was a haematocrit < 25%. Sedation and analgesia was provided with midazolam and fentanyl.

After achieving these therapeutic endpoints, MgS infusion was started at a rate of 2 g over 1 hour. For safety reasons, a sustained MAP < 65 mm Hg during infusion despite protocolized treatment constituted a reason to stop MgS infusion permanently.

The following data were recorded at baseline: general characteristics, Acute Physiology and Chronic Health Evaluation II (APACHE II) score, calculated over the first 24 hrs following ICU admission and Sequential Organ Failure Assessment (SOFA) score, calculated at study time. Directly prior to and within 10 minutes after cessation of MgS infusion systemic hemodynamic variables, sublingual SDF images and standard laboratory tests, including arterial and central/mixed venous blood gasses were obtained. ICU and hospital length of stay (LOS) and mortality were registered afterwards.

### Videomicroscopic measurements and analysis

Images of the sublingual microcirculation were obtained with SDF videomicroscopy (Microscan^®^, Microvision Medical, Amsterdam, the Netherlands) [3]. After gentle removal of saliva and other secretions by an isotonic saline-drenched gauze, the device was applied to sublingual region, avoiding pressure artifacts by establishing a threshold-image. Sequences of 20 seconds from at least three areas were recorded on hard disk using a personal computer and AVA v3.0 software (Microvision medical, Amsterdam, The Netherlands). Video clips were blindly analyzed offline by two investigators in random order to prevent coupling. Assessment of microcirculatory parameters of convective oxygen transport (microvascular flow index (MFI) and proportion of perfused vessels (PPV)), and diffusion distance (perfused vessel density (PVD) and total vessel density (TVD)) was done according to published recommendations [22]. Vessels were separated into large (mostly venules) and small (mostly capillaries) using a diameter cutoff value of 20 μm. The final MFI score is the average values of 3 × 4 quadrants. Heterogeneity index was calculated as the difference between the highest and lowest MFI, divided by the mean MFI of all sublingual sites at a single time point [23].

### Statistical analysis

Primary outcome is sublingual MFI of small vessels. We anticipated a standard deviation in difference of response of 0.5. We calculated a sample size of 14 patients to detect a absolute difference of 0.4 in a two-sided test with 0.05 type I error and a 80% probability. The Statistical Package for Social Sciences (SPSS 15.1 for Windows, Chicago, USA) was used for statistical analysis. With respect to small numbers data are presented as the median (25th-75th percentiles) and analyzed with non-parametric Wilcoxon signed rank test and Spearman's rho for correlations. A *p *value < 0.05 was considered significant.

## Results

### Patients

Fourteen patients (12 with septic shock and 2 with severe sepsis) with a median APACHE II score of 20 were enrolled, within 24 hours after the initial sepsis resuscitation. Baseline characteristics are summarized in Table 1. Hospital mortality was 57% and all patients survived the first day after MgS infusion. A source of infection was confirmed in all cases. No predefined sustained hypotension during MgS infusion was reported.

**Table 1:** Baseline characteristics

Age, yrs	62(42-74)
Gender, male/female, n	8/6

Source of sepsis, n	
Lung	4
Abdomen	7
Urinary tract	1
Skin and soft tissues	2

Hospital mortality, n(%)	8(57)

ICU length of stay, days	5(3-11)

APACHE II score	20(14-26)

SOFA score	8(5-10)

Vasopressors, n	12
Dopamine, n; μg/kg per min	5; 8.0(5.5-9.0)
Norepinephrine, n; μg/kg per min	9; 0.12(0.06-0.23)

Mechanical ventilation, n	13
PEEP, cm H2O	5(5-5)
FIO2, %	55(50-55)

Total magnesium, mEq/l	0.90(0.75-0.99)

Potassium, mEq/l	4.1(3.7-4.4)

Hematocrit, %	30(27-36)

APACHE, Acute Physiology and Chronic Health Evaluation; SOFA, Sepsis-related Organ Failure Assessment; PEEP, positive end-expiratory pressure. Data are presented as median (25^th^-75^th ^percentiles). Normal value for total magnesium is 0.9-1.2 mEq/l.

### Hemodynamic data

**Figure 1:**
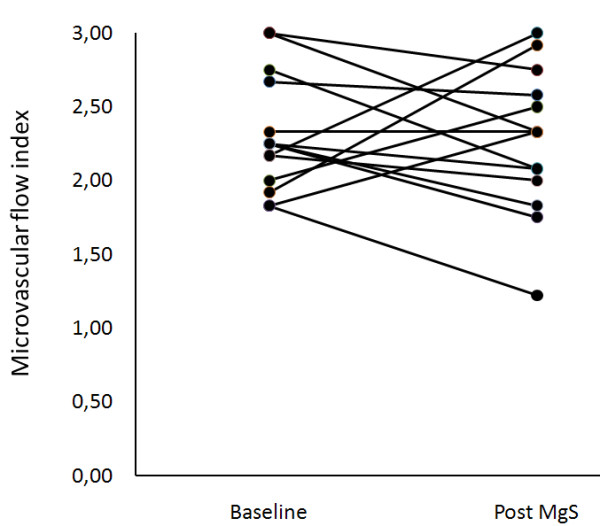
Change of microvascular flow index pre- and post magnesium sulphate infusion

**Figure 2:**
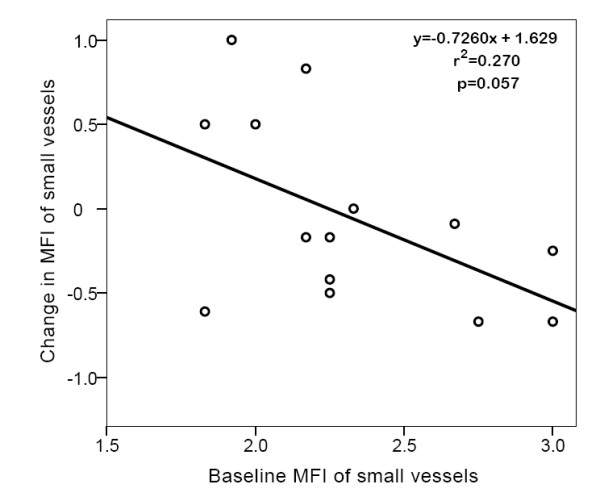
**Linear relationship between baseline microvascular flow index and change pre- and post magnesium sulphate infusion**. microvascular flow index, MFI

**Figure 3:**
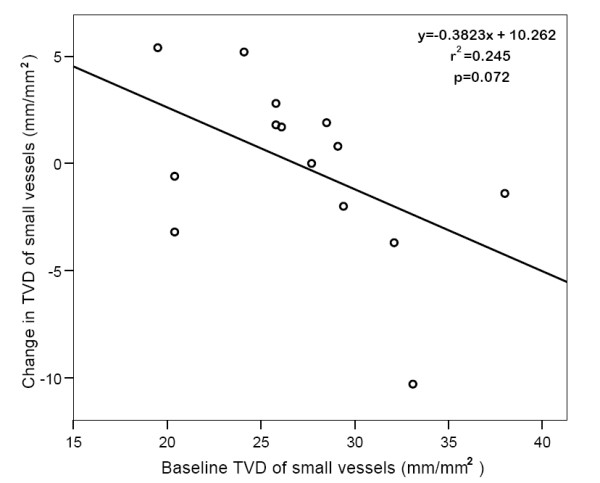
**Linear relationship between baseline total vessel density and change pre- and post magnesium sulphate infusion**. total vessel density, TVD

No significant difference in systemic hemodynamic variables was found between baseline and after MgS infusion (Table 2). Furthermore, we did not observe any significant difference pre and post MgS infusion in the primary endpoint MFI of small vessels: 2.25(1.98-2.69) vs. 2.33(1.96-2.62), p = 0.65 (Figure 1). Other variables of microcirculatory perfusion were also unaltered (Table 3). In the overall unchanged microvascular perfusion there was a non-significant trend to an inverse linear relationship between the changes of MFI of small vessels and baseline values of MFI of small vessels (y = -0.7260 × + 1.629, r^2 ^= 0.270, p = 0.057, Figure 2). We also observed non-significant trends of inverse linear relationship between the changes in TVD of small vessels and baseline values. (Figure 3). Baseline value for total Mg was 0.9(0.75-0.99); normal value for total magnesium is 0.9-1.2 mEq/l. The correlation between baseline Mg concentrations and the change in MFI pre- and post MgS infusion was non-significant (r_s _= -0.165, p = 0.67).

**Table 2:** Systemic hemodynamic data

	Baseline	MgS	*p *Value
Heart rate, beats/min	98(85-113)	94(83-115)	0.98

Mean arterial pressure, mm Hg	71(70-75)	70(67-73)	0.22

Central venous pressure, mm Hg	12(12-13)	12(11-13)	0.29

Vasopressors, n	12	12	
Dopamine, n; μg/kg per min	5; 8.0(5.5-9.0)	5; 6.0(5.5-12.0)	0.41
Norepinephrine, n; μg/kg per min	9;0.12(0.06-0.23)	9; 0.12(0.06-0.25)	0.31

Central venous oxygen saturation, n; %	9; 73(71-74)	9; 72(72-73)	0.39

Mixed venous oxygen saturation, n; %	5; 68(65-72)	5; 69(65-71)	0.72

Cardiac output, n; L/min	5; 5.4(4.6-6.8)	5; 5.5(4.8-6.4)	0.59

Lactate, mmol/L	2.7(2.4-3.6)	2.7(2.2-3.7)	0.78

pH	7.35(7.29-7.44)	7.38(7.30-7.46)	0.07

MgS, magnesium sulphate. Data are presented as median (25^th^-75^th ^percentiles).

**Table 3:** Microcirculation perfusion data

	Baseline	MgS	*p *Value
Microvascular flow index of small vessels	2.25(1.98-2.69)	2.33(1.96-2.62)	0.65

Microvascular flow index of large vessels	3.00(3.00-3.00)	3.00(3.00-3.00)	0.79

Proportion of perfused small vessels, %	81.5(78.8-89.3)	85.0(79.3-86.3)	0.64

Total vessel density of small vessels, mm/mm^2^	26.9(23.2-30.1)	27.8(24.4-29.5)	0.86

Total vessel density of others vessels, mm/mm^2^	7.4(6.2-8.9)	6.6(5.8-8.0)	0.26

Perfused vessel density of small vessels, n/mm	13.4(11.8-15.8)	13.6(11.5-15.1)	0.59

Perfused vessel density of other vessels, n/mm	4.2(3.7-4.7)	4.0(3.3-5.0)	0.97

Heterogeneity index	0.30(0.08-0.54)	0.42(0.26-0.50)	0.51

MgS, magnesium sulphate. Data are presented as median (25^th^-75^th ^percentiles).

## Discussion

To our knowledge, this is the first study in which the effect of Mg on the human microcirculation is visualized directly in patients with sepsis. The main finding of our study is that MgS infusion had no effect on both sublingual microvascular perfusion and systemic hemodynamic variables in this setting. This is not in line with previous observations, that evaluated the effect of MgS on microcirculatory perfusion under non-septic conditions. In vivo plethysmography of the forearm in healthy volunteers during intra-arterial MgS infusion revealed a marked increased in blood flow. In an animal model with direct in-vivo microscopy of the microcirculation, both topical and intravenous application of Mg-compounds demonstrated vasodilatory effects in mesenteric arterioles (10-20 μm) and venules (15-30 μm), that could not be blocked by cyclo-oxygenase inhibition [24]. This effect was also observed during catecholamine-induced vasoconstriction [25].

The discrepancy between these results and our observations may in part be explained by a difference in technique. Plethysmography incorporates artiolar, capillary and venular blood flow. In sepsis however, a heterogeneous distribution of altered capillary blood flow is observed, often despite maintenance of both artial and venular blood flow. In contrast to plethysmography, SDF enables discrimination between these different vascular compartments. Furthermore, sublingual SDF imaging may not necessarily reflect microvascular perfusion abnormalities in vascular beds of other organs [26]. Whether the sensitivity of software-supported analysis of TVD of vessels equals to direct diameter measurements of vessels during in-vivo microscopy remains to be established and is very sensitive to adequate focus.

The classical response to overcome microvascluar perfusion abnormalities in shock has always been to raise arterial pressure by means of vasopressors, in order to increase the net perfusion pressure over the microvascular compartment. However, according to physiological theory, the drop in perfusion pressure prior to the microcirculation is equal to the increase in artiolar resistance, thus reducing its pro-microcirculatory flow effect [27]. Recent studies indeed failed to demonstrate a pro-microcirculatory effect after a stepwise increase of blood pressure with norepinephrine [28,29]. Although the alternative concept of vasodilation to recruit microcirculatory perfusion in sepsis is tempting [30], up till now no clinically available vasodilators with pro-microcirculatory characteristics have been discovered. Despite promising results in a small group of patients [9], nitroglycerin showed no effect on microcirculatory perfusion in a randomized placebo controlled trial in patients with severe sepsis and septic shock [10]. Both the latter study and the presented trial have in common that a vasodilator was infused on top of a strict resuscitation protocol, making hypovolemia unlikely. Even under well-descript vasodilatory effects of epidural anesthesia, hypervolemic hemodilution completely blunted microcirculatory changes in flow and capillary density of the vaginal mucosa [31]. The absence of effect of a vasodilator in this setting, both on macro- and microcirculatory variables, may be in line with maximal pre-capillary smooth muscle relaxation prior to the start of the intervention. This is particularly of note since, unlike nitroglycerin, MgS has an additional non-endothelium dependent pathway to exert its vasodilatory capacities, by a direct action on vascular smooth muscle relaxation as a calcium antagonist [15].

Our study has a number of limitations. Due to the open label setting and relatively small numbers a modest effect of MgS infusion on microcirculatory perfusion may have remained unnoticed. Baseline MFI was decreased despite resuscitation, but only to a moderate extend. Although this may have blunted a potential pro-microcirculatory effect of MgS, we only observed a non-significant and weak correlation between baseline MFI and change in MFI in response to therapy. It must be stated however, that post-hoc linear regression analysis itself is limited by the narrow range of variables. We deliberately chose this strategy, since we consider it key not to replace therapeutic macro-hemodynamic strategies by micro-hemodynamic strategies, but to add micro-hemodynamic strategies in case of remaining microcirculatory abnormalities, despite optimal macro-hemodynamic resuscitation. In our study we used a continuous fixed dose of MgS without bolus due to safety reason, since some data show, that higher doses of MgS may inhibit catecholamine release [32,33]. Endothelium-independent vasodilation is associated with venous Mg concentrations, but the endothelium-mediated vasodilatory pathway of Mg appears to be activated, irrespective of its plasma levels [14]. In the present study there was no correlation between baseline Mg concentrations and the change in MFI pre- and post MgS infusion. We failed to report plasma Mg concentrations after infusion. The observation period after the intervention was without delay. This may have been too short to allow microcirculatory alterations to respond to the intervention. On the other hand, longer observation periods in an open label setting carry a considerable risk of improvement over time, irrespective of the intervention itself. Therefore, we cannot rule out that a higher dose, a different loading regiment, suppletion to a pre-defined plasma Mg concentrations or a longer post-intervention observation period may have revealed more potential of MgS infusion for the improvement of microcirculatory perfusion in sepsis.

## Conclusion

In the setting of severe sepsis and septic shock sublingual microcirculatory alterations were observed despite fulfillment of sepsis resuscitation guidelines. After infusion of a limited and fixed dose of MgS, microcirculatory perfusion did not improve over time.

## List of abbreviations

OPS: orthogonal polarization spectral; SDF: sidestream dark field; NO: nitric oxide; Mg: magnesium; MgS: magnesium sulphate; MAP: mean arterial pressure; SvO_2_: mixed venous saturation; S(c)vO_2_: central venous saturation; APACHE: Acute Physiology and Chronic Health Evaluation; SOFA: Sequential Organ Failure Assessment; LOS: length of stay; MFI: microvascular flow index; PPV: proportion of perfused vessels; PVD: perfused vessel density total vessel density (TVD)

## Competing interests

The authors declare that they have no competing interests.

## Authors' contributions

AP conceived the study, performed SDF imaging and wrote the first draft; NV analyzed SDF images; VP participated in the design of the study; MK was responsible for data acquisition; CB performed statistical analysis and wrote the final manuscript. All authors read and approved the final manuscript.

## Pre-publication history

The pre-publication history for this paper can be accessed here:

http://www.biomedcentral.com/1471-2253/11/12/prepub
